# Qilin Is Essential for Cilia Assembly and Normal Kidney Development in Zebrafish

**DOI:** 10.1371/journal.pone.0027365

**Published:** 2011-11-15

**Authors:** Jade Li, Zhaoxia Sun

**Affiliations:** Department of Genetics, Yale University School of Medicine, New Haven, Connecticut, United States of America; Universität Heidelberg, Germany

## Abstract

Defects in the cilium, a once thought vestigial organelle, have recently been implicated in many human diseases, including a number of cystic kidney diseases such as polycystic kidney disease (PKD), Bardet Bieldl Syndrome, and Meckel-Gruber Syndrome. In a forward genetic screen, *qilin* was identified as a novel gene important in the pathogenesis of kidney cysts in zebrafish. In this paper we characterized *qilin^hi3959A^* mutant's phenotypes in detail, investigated cilia formation in this mutant and performed structural and functional analysis of the Qilin protein. Results reveal Qilin's essential role in cilia assembly and maintenance in multiple organs, including the kidney, the lateral line organ, and the outer segment of the photoreceptor cell. In addition, rescue experiments suggest that defective pronephric cilia correlate with the formation of kidney cysts in *qilin^hi3959A^* mutants. Further, genetic analysis suggests that *qilin* interacts with multiple intraflagellar transport (IFT) complex B gene*s*, which is supported by the striking phenotypic similarities between *qilin^hi3959A^* and IFT complex B mutants. Finally, through deletion analysis we provide evidence that the well-conserved N-terminus and the coiled-coil domain of Qilin are both essential and sufficient for its function. Taken all the observations together, we propose that Qilin acts in a similar role as IFT complex B proteins in cilia assembly, maintenance and kidney development in zebrafish.

## Introduction

The cilium, an organelle projecting from the cell surface, had long been believed to be vestigial in vertebrates; however, in the past decade it has been shown to play a critical role in both vertebrate physiology and development. Defects in cilia are being linked to an increasing list of human diseases, including polycystic kidney disease (PKD), Bardet-Biedl Syndrome (BBS) and Joubert syndrome, as well as developmental defects such as situs inversus and polydactyl [1]–[11]. Despite the growing awareness of the functional importance of cilia, our understanding of the regulation of cilia biogenesis and maintenance remains incomplete.

In a forward genetic screen in zebrafish, a group of cystic kidney mutants were identified [12]. Consistent with a central of cilia in PKD pathogenesis, three of the identified genes encode components of complex B of intraflagellar transport (IFT) particles. First identified in the green algae *Chlamydomonas*, IFT particles are multi-protein complexes believed to carry cargos essential for cilia biogenesis, maintenance and signaling [13], [14]. They are composed of complex A and complex B subunits, with complex A more associated with retrograde transport [15], [16] and complex B involved in anterograde transport [13], [17]. Interestingly, despite phenotypic similarities, some mutants isolated in the screen exhibited cilia biogenesis defects while others were able to assemble cilia [12]. *Qilin* (also later named as *Cluap1* as its encoded protein was identified as a Clusterin-associated protein, [18]) was a novel gene identified in this screen, and *qilin^hi3959A^* mutants develop kidney cysts but are capable of cilia assembly [12]. However, although Qilin was not among the IFT particle components biochemically purified from *Chlamydomonas*, subsequent studies involving Qilin homologues in other organisms suggest a link between Qilin and cilia. In *C. elegans*, the Qilin homologue DYF-3 was observed to move along the cilia at exactly the same biphasic anterograde rate as those reported for known IFT particles and IFT motors in *C. elegans,* providing strong evidence that DYF-3 is part of the IFT machinery [19], [20]. In addition, *dyf-3* mutants develop truncated sensory cilia, suggesting that *dyf-3* plays a role in cilia formation or maintenance in *C. elegans* [19], [21]. A connection between Qilin and cilia is also implicated by the identification of its human homologue as part of the human cilia proteome, as well as the observation that its homologue in *Chlamydmonas* is highly upregulated during flagella regeneration [22], [23].

Although implicated in cilia biogenesis, the precise role of Qilin in cilia formation, maintenance and embryonic development, particularly in vertebrates, remains unclear. In this study, we characterized the zebrafish *qilin^hi3595A^* mutant in detail. We show that *qilin* is a maternally supplied gene and the maternal contribution masks the essential function of Qilin in cilia biogenesis and maintenance during early development in zebrafish. Further, in addition to almost identical morphological phenotypes, *qilin^hi3595A^* mutants display similar cilia biogenesis defects as IFT complex B mutants. Moreover, we provide evidence that *qilin* genetically interacts with multiple IFT B complex genes. Together, these results suggest that Qilin functions in the same pathway as IFT B complex genes in cilia biogenesis. Finally, through deletion analysis we show that the N-terminus together with the coiled-coil domain of the Qilin protein is both necessary and sufficient for Qilin's function.

### Highlights

- *qilin* is essential for cilia formation and maintenance in zebrafish
- *qilin* functions in similar processes as intraflagellar transport (IFT) genes
- N-terminal and coiled-coil domains of Qilin are essential and sufficient for its functions

## Materials and Methods

### Zebrafish husbandry

Standard protocols were used for maintaining zebrafish colonies. Embryos were obtained through natural spawning. All lines were maintained in the TAB background.

All zebrafish works have been conducted according to protocols approved by Institutional Animal care and Use Committee (IACUC) of Yale University (Protocol number: 2009–10778).

### RT-PCR

RNA was extracted from embryos at different developmental stages using Trizol reagent (Invitrogen) according to manufacturer's instructions. Total RNA was reverse-transcribed using an oligo-dT primer and the Superscript II RT-PCR system (Invitrogen). Subsequent Qilin-specific PCR was performed with the following primers: 5′-TACAACTAAAACGGTGACAGT-3′ and 5′-AACCCTCTCAAACTCACAAATTAAC-3′.

### Genotyping *qilin^hi3959A^* mutants

To genotype progeny from *qilin^hi3959A^* carrier in-crosses, three primers were used. One primer is specific to the proviral insertion, close to the insertion site (5′-ACTTGTGGTCTCGCTGTTC-3′). The rest two (5′-GTGACGAACACAGCAACAGACG-3′ and 5′-CCAGTAAACACACAACTGTCACC-3′) are specific for genomic regions flanking the proviral insertion. In the absence of the proviral insertion, amplification would take place between the genomic pair. In contrast, the presence of the 10 kb proviral insertion will disrupt the amplification between the genomic pair. Instead, amplification would occur between the proviral-specific primer and one of the genomic primers.

### Histological analysis

Embryos were fixed in Bouin's fixative overnight at room temperature, washed three times in PBS with 0.1% Tween-20 (PBT), embedded in JB-4 resin (Polysciences) following manufacturer's instructions and cut at 4 µm with a microtome. Slides were then stained with hematoxylin and eosin.

### Generation of Qilin constructs

The *qilin* coding sequence was PCR amplified from a zebrafish cDNA pool and cloned into the pCS2+ vector. pCS2-*qilin* tagged with *eGFP* on the N-terminus end was generated via PCR cloning. Different *qilin* deletion constructs were generated via PCR cloning.

### In situ hybridization

Embryos were fixed in diluted formalin (1∶2.7 in PBT) at room temperature for an hour or at 4°C overnight. Digoxigenin-UTP labeled RNAs synthesized in vitro were used as probes. Alkaline phosphatase-coupled anti-digoxigenin (Roche) was used to localize hybridized probes. NBT/BCIP (Roche) was used as the chromogenic substrate to produce blue precipitates.

### Assay for rescue activity

mRNA was synthesized in vitro using the mMESSAGE mMACHINE kit (Ambion) following the manufacturer's instructions. 174 pg mRNA of *eGFP-qilin*, as well as other deletion constructs were injected into zebrafish embryos. Embryos were scored for body curvature at 2 dpf (days post-fertilization), and for pronephric cysts at 4–5 dpf.

### Immuno-staining

Embryos were anaesthetized with MESAB and fixed in Dent's fixative (80% methanol and 20% DMSO). The following antibodies were used: mouse monoclonal anti-ϒ tubulin antibody (1∶200 dilution, Sigma T5326), mouse monoclonal anti-acetylated tubulin antibody (1∶5000 dilution, Sigma clone 6-11b-1), rabbit polyclonal anti-Scorpion antibody (1∶2000 dilution, [24]) and rabbit polyclonal anti-Cdh17 (1∶200, [24]). Secondary antibodies from Jackson Immuno Research Laboratories Inc were used at 1∶200 dilutions. DAPI (Invitrogen D3571) was used at 1∶10,000 dilution.

### Morpholino Oligos

Morpholino oligonucleotides were purchased from Gene Tools and injected into zebrafish embryos at the one- to four-cell stages. 5′-CATGATTGCTGTCCTTTAATCCAGT-3′ was used to block the translation of *qilin*. Previously described morpholino oligo was used to block the translation of *ift172* [25]. 5′-GGAGGTAATAGTGTGTGTCTACGTG-3′ was used to block the translation of *ift27* and 5′- GGACGTAATACTGTCTGTGTACCTG -3′ was used as the mismatch control.

### Statistical Analysis

Microsoft Excel was used to derive standard deviation and to perform student's t-tests.

## Results

### *qilin^hi3959A^* mutants develop kidney cysts and ventrally curved body axis

**Figure 1:**
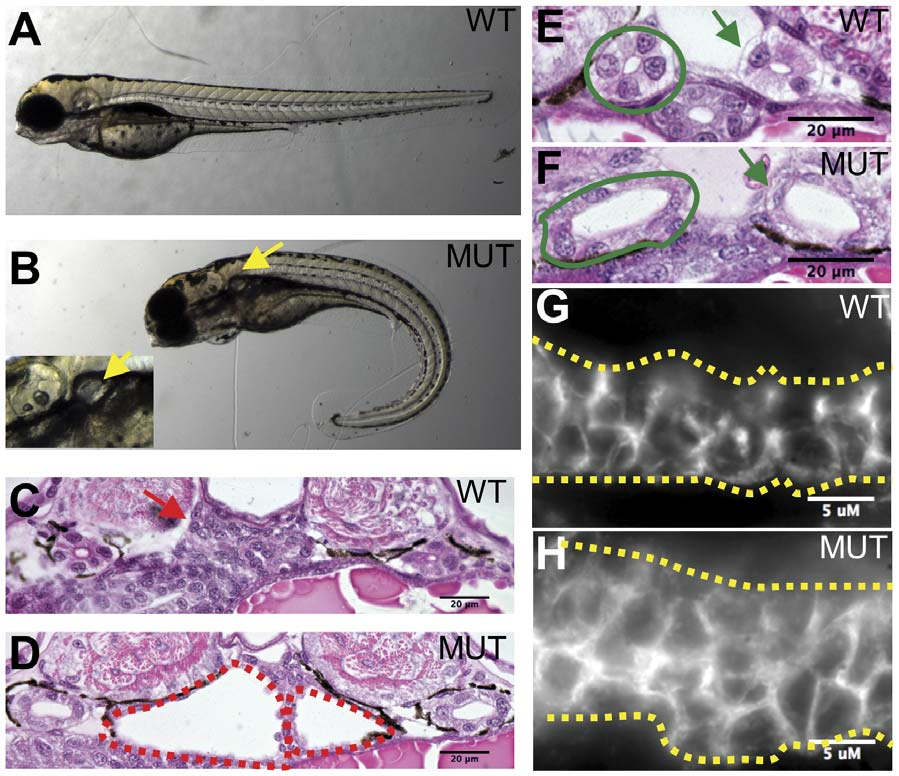
*qilin^hi3959A^* mutant develops body curvature and kidney cysts. (**A, B**) Side view of embryos at 3 dpf. Inset in B is a zoomed in view of the kidney cyst in the mutant. (**C**–**D**) Cross section through the glomerular-tubular region of embryos at 5 dpf. Red arrow in C points to the fused glomeruli, while red dotted lines in D outline the cysts. (**E**–**F**) Cross sections of the duct of embryos at 5 dpf. Solid green lines outline the duct. Green arrows point to the duct. (**G**–**H**) Side view of the pronephric duct in whole-mount embryos at 5 dpf stained with Cdh-17. Yellow dotted lines outline the duct. WT: wild type; MUT: mutant. Scale bars in C–F: 20 µm, in G and H; 5 µm.

*qilin^hi3959A^* was identified in an insertional mutagenesis screen for cystic kidney in zebrafish [12]. Similar to other group II mutants identified in this screen, *qilin^hi3959A^* mutants develop ventrally curved bodies, visible at the end of 1 dpf, followed by bilateral kidney cysts, detectable by 2 dpf (Fig. 1A, B). In addition, by 5 dpf, we observed pericardial edema in 40–60% of *qilin^hi3959A^* mutants (data not shown).

To analyze kidney cysts in greater detail, we examined the kidney in *qilin^hi3959A^* mutants using histological analysis. Cross sections of *qilin^hi3959A^* mutant embryos at 5 dpf revealed large, bilateral cysts in the glomerular-tubular region not seen in wild type sibling embryos (Fig. 1C, 1D). In addition, mutant kidney ducts are grossly enlarged compared to their wild type siblings (Fig. 1E, 1F). Kidney duct dilation in *qilin^hi3959A^* mutants was further confirmed by immunofluorescent staining of whole-mount embryos using a kidney epithelium-specific marker, Cdh17 (Fig. 1G, 1H).

### Disruption of *qilin* is responsible for phenotypes observed in hi3959A mutants

**Figure 2:**
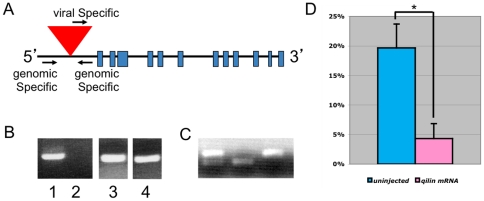
*hi3959A* is a zygotic null allele of *qilin*. (**A**) Graphic representation of *qilin* gene. Blue squares represent exons. Red triangle indicates the proviral insertion site. Arrows indicate the direction and location of the genotyping primers used. (**B**) *qilin* transcript in embryos at 5 dpf shown by RT-PCR of cDNA from *hi3959A* embryos (lane 2) and wild type siblings (lane 1). Lane 3 and 4 are beta-actin loading controls for *hi3959A* (Lane 4) and wild type siblings (Lane 3). (**C**) Genotyping PCR of embryos from *hi3959A* carrier in-crosses injected with *eGFP-qilin* mRNA. All embryos genotyped exhibited wild type phenotypes. Lower band is specific for the mutant allele, while the upper band is specific for the wild type allele. From left to right, the three embryos are heterozygous, homozygous mutant and homozygous wild type, respectively. (**D**) Graph displaying percent embryos with the curved body phenotype in *hi3959A* carrier-in crosses that are uninjected (n = 3, with an average of 140 embryos per experiment) and injected with *eGFP-qilin* mRNA (n = 3, with an average of 120 embryos per experiment). * p< 0.05.

*qilin* is a novel gene with twelve exons (Fig. 2A). In *hi3959A* mutants, a proviral insertion is located in the 5′ UTR of the gene (Fig. 2A). RT-PCR using 2 dpf embryos revealed that full-length *qilin* transcript is absent in *hi3959A* mutants (Fig. 2B).

To provide further support for our hypothesis that disruption of *qilin* resulted in the body curvature and kidney cyst phenotype observed in *hi3959A* mutants, we designed morpholino oligo against the AUG translational start site of *qilin*. Wild type embryos injected with *qilin* morpholino displayed similar phenotypes to those observed in the *hi3959A* mutants, with ventrally curved bodies and kidney cysts (data not shown).

Finally, we performed rescue experiment using mRNA synthesized in vitro. Embryos from *hi3959A* heterozygous carrier in-crosses were injected with *eGFP-qilin* mRNA, encoding Qilin tagged with eGFP at the N-terminus. In three different experiments, an average of 4% of the injected embryo displayed the body curvature phenotype, compare to 20% of the uninjected siblings (Fig. 2D). To confirm that Qilin over-expression rescues the *qilin^hi3959A^* mutant phenotype, we genotyped phenotypically wild-type embryos from those injected with the *eGFP-qilin* mRNA. Of seventeen embryos genotyped, three were homozygous for the *hi3959A* proviral insertion (Fig. 2C). The rescuing capacity of untagged *qilin* mRNA is similar to that of *eGFP-qilin* (data not shown). Taken together, these data indicate that the *hi3959A* mutant phenotypes are due to the lack of wild type Qilin.

### *qilin* is ubiquitously and maternally expressed

**Figure 3:**
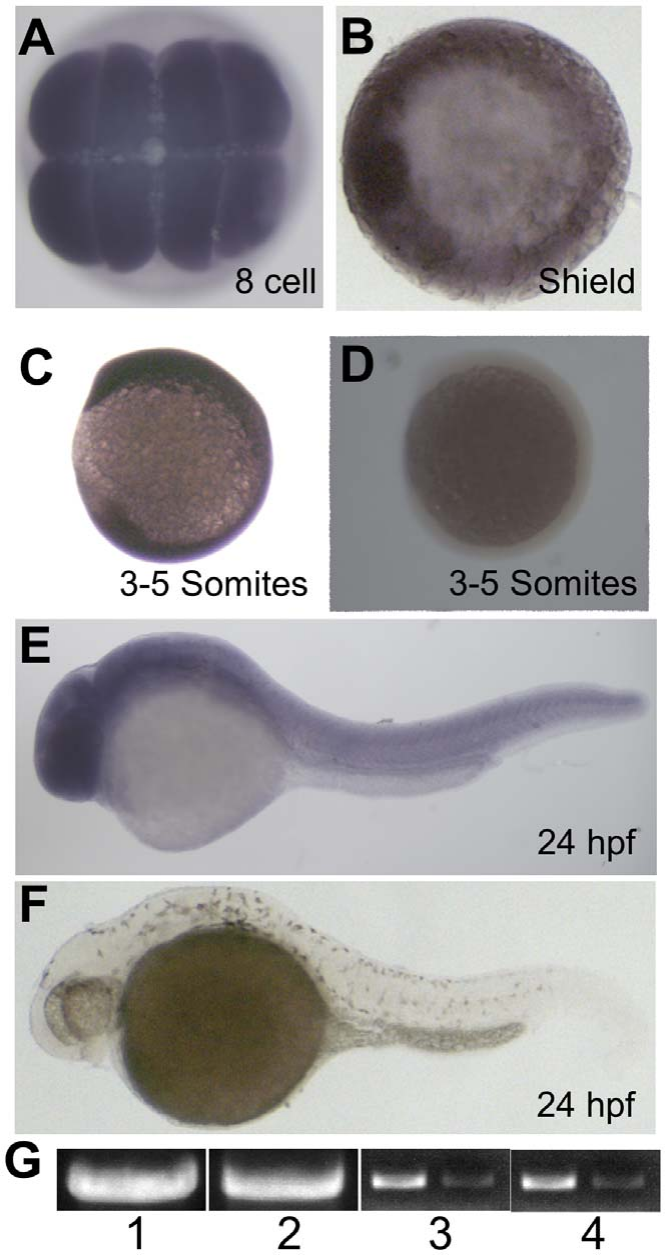
*qilin* is expressed maternally and ubiquitously. (**A**–**F**) In situ hybridization for *qilin* on embryos at the 8- cell stage (A), the shield stage (B), the 3–5 somites stage (C), and the 24 hours-post-fertilization (hpf) stage (E). D and F are sense controls at the 3–5 somites stage (D) and the 24 hpf stage (F). (**G**) *qilin* transcript shown by RT-PCR from 16 cell (Lane 1) and 2 dpf (Lane 2) wild type embryos. Lane 3 and 4 are *elf1a* loading controls for 16-cell (Lane 3) and 2 dpf (Lane 4) samples with matching dilutions of cDNA used in PCR reactions.

To examine the function of *qilin* during embryonic development, we first analyzed the expression profile of *qilin* using in situ hybridization. Results showed that *qilin* mRNA is distributed ubiquitously throughout the embryo across different developmental stages, from as early as the 8-cell stage to 24 hpf (Fig. 3A–F). The specificity of the result is verified by the complete lack of signal in embryos hybridized with a sense control probe (Fig. 3D, F). RT-PCR analysis further verified the presence of *qilin* transcript at both the 16-cell and the 2 dpf stages in wild-type embryos (Fig. 3G). In zebrafish, zygotic expression initiates when embryos reach approximately the one thousand-cell stage. Therefore these observations suggest that *qilin* mRNA is maternally supplied.

### *qilin^hi3959A^* mutants exhibit cilia biogenesis defects in the pronephric duct

**Figure 4:**
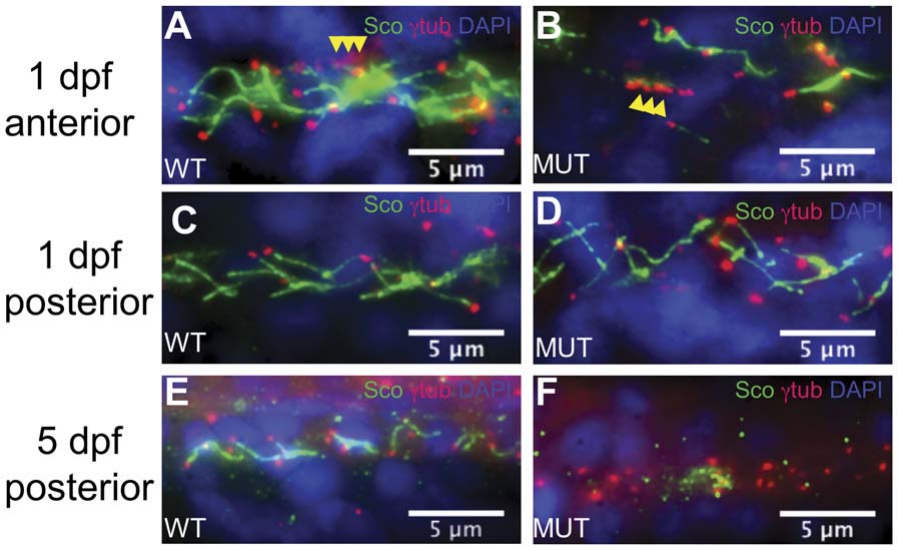
Pronephric cilia in *qilin^ hi3959A^* mutants are defective. (**A**–**D**) Epifluorescent projections showing the pronephric cilia in a wild-type sibling (A, C) and a mutant (B, D) at the 1 dpf; in both anterior (A, B) and posterior (C,D) portions of the duct. Yellow arrowheads in A and B point to rows of basal bodies in MCCs. (**E, F**) Epifluorescent projections showing the pronephric cilia in a wild-type embryo (E) and a mutant (F) at 5 dpf in the posterior portion of the duct. All embryos were stained with anti-γ-tubulin (red), anti-Sco (green), and DAPI (blue).

To investigate whether Qilin is required for cilia assembly or maintenance, we examined cilia morphology at various developmental time points in the pronephric duct of *qilin^hi3959A^* mutants using the anti-Sco/Arl13b antibody, a cilia-specific marker we established in a previous study [24], [26]. In the pronephric duct, there are two populations of ciliated cells: the multi-ciliated cells (MCCs) that produce bundled cilia, and the single-ciliated cells (SCCs) that display only a single cilium per cell [27], [28]. Both populations are present in the anterior to middle regions of the pronephric duct, while only the SCC population is present in the posterior region of the duct. On 1 dpf, cilia in the anterior pronephric duct of *qilin^hi3959A^* mutants are already visibly defective (Fig. 4A, B): while individual cilia from SCCs are still present in the mutants, cilia bundles from MCCs are absent. Consistently, cilia of the posterior pronephric duct, which contains SCCs but not MCCs, appear unaffected in *qilin^hi3959A^* mutants (Fig. 4C, D). In comparison, by 5 dpf, the entire pronephric ducts of *qilin^hi3959A^* mutants lack cilia, whereas the ducts of wild type embryos have abundant cilia (Fig. 4E, F). Cilia displayed by SCCs that were initially present in the mutants are no longer detectable (Fig. 4E, F). Because bundled cilia never form in *qilin^hi3959A^* mutant, Qilin appears to be necessary for the assembly of cilia in MCCs. Meanwhile, since single cilia develop normally but become defective over time, Qilin seems to be required for the maintenance, not initial biogenesis, of cilia in SCCs.

### Formation of single cilia in the pronephric duct precedes the formation of cilia bundles in multi-ciliated cells

**Figure 5:**
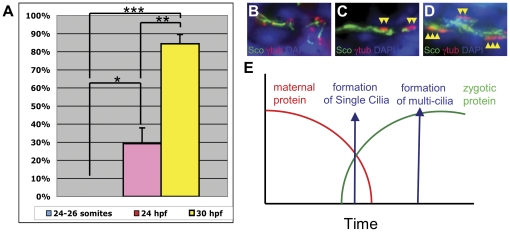
Single cilia form earlier in development than multi-cilia in the pronephric duct. (**A**) Graphical representation of multi-cilia observed in embryos at 24 somite, 24 hpf, and 30 hpf. Single cilia were observed at all time points analyzed. Bars represent percentage of embryos that developed multi cilia in the pronephric ducts. Each bar represents data from three independent experiments with at least 8 embryos each. (**B**–**D**) Representative images of cilia at 24-somite (B), 24 hpf (C) and 30 hpf (D). Yellow arrowheads point to basal bodies of multi-cilia. (**E**) A model of how different developmental timing of single cilia and multi-cilia could contribute to the pronephric cilia phenotypes observed in *hi3959A* mutants.

The differential cilia phenotypes in the kidney duct of qilin^hi3959A^ mutants may suggest a cell-type specific function of Qilin. Alternatively, in light of the maternal contribution of Qilin transcript, this phenotypic difference may simply be caused by differential developmental timing of the formation of single cilia and bundles of multi-cilia in the pronephric duct. To test this hypothesis, we performed a careful time course analysis on the formation of cilia in the pronephric duct. At the 24-somite stage, in none of the wild-type embryos analyzed from three independent experiments can bundled cilia be detected, while individual cilia are clearly visible (Fig. 5A, B). At 24 hpf, single cilia are visible in all analyzed wild-type embryos, but multi-cilia are only detected in 22% of the embryos (Fig. 5A, C). At 30 hpf, the percent of embryos with detected multi-cilia increases to 75% (Fig. 5A, D). These observations suggest that single cilia indeed develop earlier than multi-cilia in the developing pronephric duct, consistent with the hypothesis that maternally contributed qilin mRNA was sufficient to support the formation of single cilia, but not bundled multi-cilia (Fig. 5E).

### *qilin ^hi3959A^* mutants exhibit cilia biogenesis defect in multiple sensory organs

**Figure 6:**
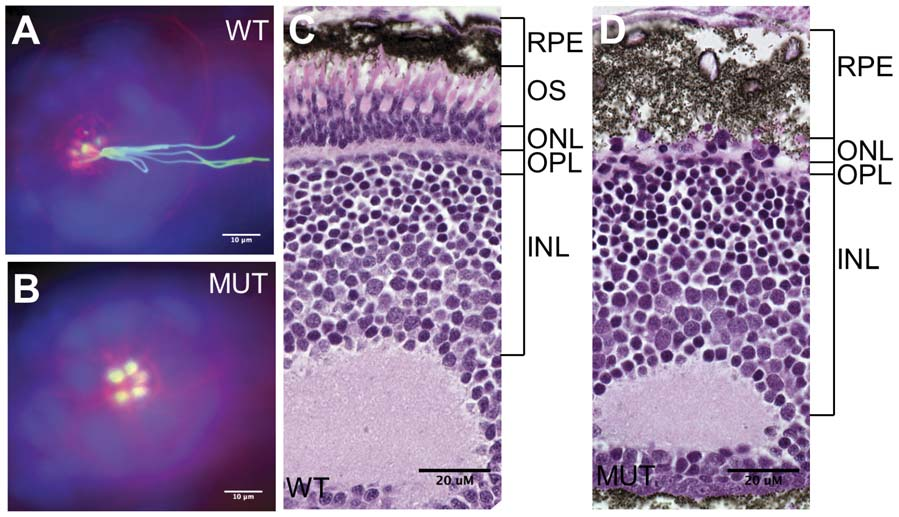
Sensory cilia in *qilin^ hi3959A^* mutants are defective. (**A**–**B**) Epifluorescent projections showing the lateral line organ in a wild type (A) and a mutant embryo (B) at 3 dpf. Embryos were stained with rodamine-phalloidin (red), anti- acetylated tubulin (green) and DAPI (blue). Scale bars: 10 µm. (**C**–**D**) The absence of the outer segment of photoreceptors in the eye as shown through cross sections of a wild type (C) and a mutant embryo (D) at 5 dpf. WT: wild type; MUT: mutant; RPE: retinal pigment epithelium; OS: outer segment; ONL: the outer nuclear layer; INL: the inner nuclear layer; OPL: the outer plexiform layer. Scale bars: 20 µm.

Given our finding that Qilin is important in the assembly and maintenance of both populations of pronephric cilia, we were interested in whether Qilin is required for the assembly of cilia in other ciliated cells. We chose to examine ciliated organs formed later in development to eliminate the possibility of maternally contributed *qilin* masking its requirement in cilia assembly. The lateral line organ usually displays long cilia in wild-type embryos by 2 dpf (Fig. 6A). In *qilin^hi3959A^* mutants, however, there are no visible cilia in this organ (Fig. 6B). Notably, the structure of the lateral line organ structure is not affected in *qilin^hi3959A^* mutants, suggesting that the lack of Qilin results in a very specific defect in cilia assembly.

*qilin^hi3959A^* mutants also display cilia biogenesis defects in the eye. In the retina, the outer segments (OSs) of photoreceptor cells are modified cilia, which appear as a lightly stained layer between the retinal pigment epithelium (RPE) and the out nuclear layer (ONL) in histological sections (Fig. 6C). We found that in *qilin^hi3959A^* mutants, the entire outer segment is missing and the number of nuclei in the outer nuclear layer, which is comprised of the cell bodies of photoreceptor cells, is much reduced. By contrast, the inner nuclear layer (INL) is intact (Fig. 6D). Together, these results suggest that Qilin is required for cilia biogenesis and maintenance in multiple sensory organs in zebrafish.

#### qilin genetically interact with ift172 and ift27

**Figure 7:**
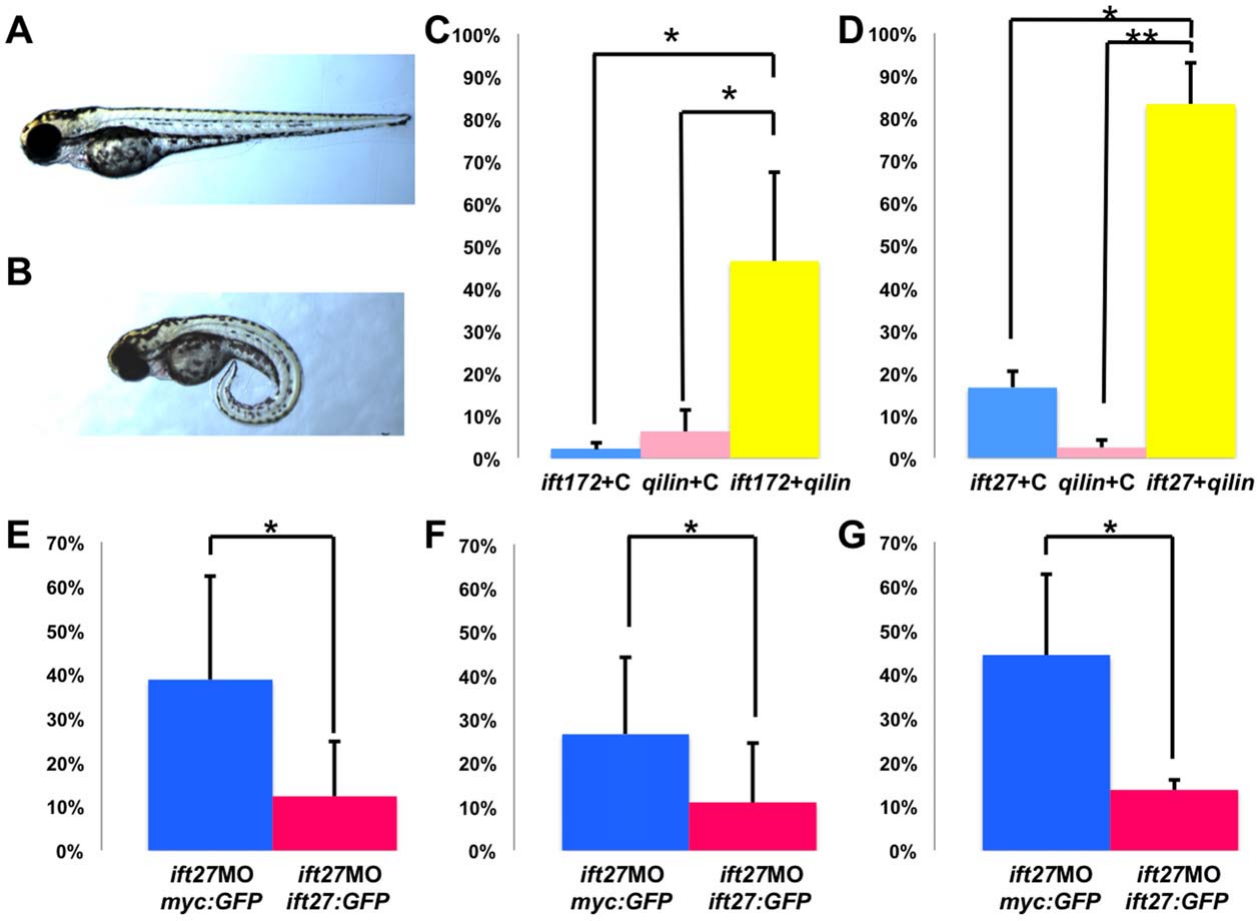
*qilin* genetically interacts with *ift172* and *ift27*. (**A**–**B**) Side view of a representative uninjected control embryo (A) and a phenotypic embryo injected with *qilin* and/or *ift172*, *ift27* morpholino (B) at 2 dpf. (**C, D**) Graph displaying the percent of embryos that develop curved bodies. In C, embryos are either injected with *qilin* morpholino and the control morpholino; *ift172* morpholino and the control morpholino; or *qilin* morpholino with *ift172* morpholino. In D, embryos are either injected with *qilin* morpholino and the *ift27* mismatched-control morpholino; *ift27* morpholino and the *ift27* mismatched-control morpholino; or *qilin* morpholino with *ift27* morpholino. (**E**–**G**)Graphical representation of the effectiveness of *ift27:GFP* in rescuing body curvature (E), kidney cysts (F), and laterality defects (G) observed in *ift27* morphants. Embryos are either injected with *ift27* morpholino and *myc:GFP* mRNA (blue bars), or *ift27* morpholino with *ift27:GFP* mRNA (pink bars). Laterality defects in (G) is presented as the percentage of embryos developed hearts positioned on the right or center. N = 3 for all experiments, with at least 40 embryos per experiment per condition. *: p< 0.05, **: *p*< 0.01. MO: morpholino.

qilin^hi3959A^ mutants display many of the phenotypes reported for IFT complex B mutants, including similar body curvature and kidney cyst formation [12]. On the cellular level, *qilin^hi3959A^* mutants also show similar cilia phenotypes to that of IFT complex B mutants, but distinct from other cystic kidney mutants in zebrafish [24], [25], [26]. These observations led us to test whether *qilin* genetically interacts with genes encoding IFT complex B components. Using a morpholino-based assay we first titrated morpholino oligos against *qilin*, *ift172* and *ift27* to their suboptimal dosages and then combined two different morpholinos to test whether they act synergistically by assessing the percentage of embryo developing the body curvature phenotypes (Fig. 7A, 7B). Specifically, when wild-type embryos were injected with 2.0 ng *ift172* morpholino together with 6.7 ng control morpholino, or 6.7 ng *qilin* morpholino together with 2.0 ng control morpholino, only 2% and 6.7% displayed the body curvature. However, when wild-type embryos were injected with 2.0 ng *ift172* morpholino together with 6.7 ng q*ilin* morpholino, 46.4% developed body curvature (Fig. 7C).

The same assay was performed between *qilin* and another IFT complex B gene, *ift27*. We designed a morpholino oligo against the translational initiation site of *ift27*. At the optimal dosage of 8.0 ng, this oligo causes the development of body curvature, kidney cyst, and laterality defect as shown by the position of the heart. Importantly, all three phenotypes can be rescued by expressing a full length *ift27 *mRNA in the morphants (Fig. 7 E–G), validating the specificity of the *ift27* morpholino. When wild-type embryos were injected with the suboptimal 4.0 ng *ift27* morpholino together with 6.7 ng of a general control morpholino, or 6.7 ng *qilin* morpholino together with 4 ng *ift27* mismatch-control morpholino, only 14% and 3.0% of the embryos developed the curved body phenotype, respectively. However when wild-type embryos were injected with 4.0 ng *ift27* morpholino together with 6.7 ng *qilin* morpholino 88.2% of the embryos developed body curvature (Fig. 7D). These results, together with the phenotypic similarities between *qilin^hi3595A^* mutants and multiple IFT complex B mutants, support our hypothesis that Qilin functions with the IFT B complex in similar processes.

### Structural and functional analysis of the Qilin protein

**Figure 8:**
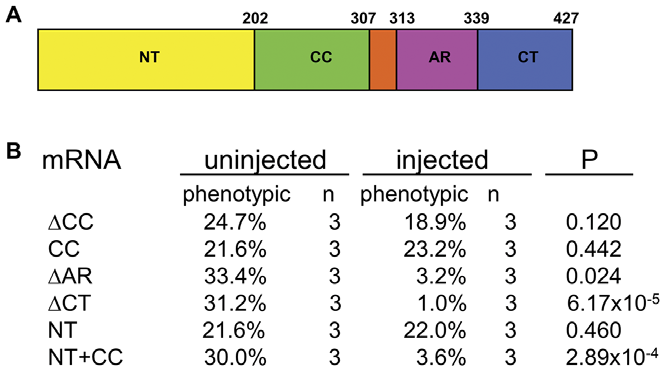
The N-terminus together with the coiled-coil domain is sufficient for Qilin '**s function. **(**A**) Diagram of Qilin structure. NT: N-terminal region; CC: coiled coil domain; AR: aspartic acid rich region; CT: C-terminal tail. Numbers are amino acid coordinates in the protein. All constructs were tagged with eGFP at the N-terminus. (**B**) Table summarizing the rescuing ability of different deletion constructs assayed in three independent experiments for each construct, with at least 45 embryos per sample per experiment.

The Qilin protein (Q7ZVC2.2) is predicted to have a coiled-coil domain and an aspartic acid rich domain (Fig. 8A). The zebrafish Qilin is fairly conserved with its mouse and human homologue with an overall identical percent of 64% and 62% respectively (Table S1). The N-terminus and the coiled-coil domain are especially well conserved, with an identical percent of 80% and 79% respectively. To delineate the functional significance of the structural features of Qilin, we generated a series of deletion constructs and tested their ability to rescue *qilin^hi3595A^* mutant phenotypes. To ensure that the deletion proteins were stably expressed, we tagged each of them with eGFP to the N-terminus and verified the presence of eGFP signal in ensuring assays. Importantly, similarly tagged full length Qilin was able to rescue *qilin^hi3595A^* mutants (Fig. 2).

We started with the construct that encodes Qilin without the coiled-coil domain (eGFP-ΔCC). *qilin^hi3595A^* heterozygote in-crosses injected with ΔCC mRNA developed curvature and kidney cysts at an average of 18.9% from three experiments, not significantly different from uninjected embryos where an average of 24.7% of the embryos developed curvature and kidney cysts (Fig. 8B), suggesting that the coiled-coil domain is necessary for Qilin's functions. We further tested if the coiled-coil domain itself is sufficient to rescue the *qilin^hi3595A^* mutant phenotype by over-expressing the coiled-coil domain (eGFP-CC) in embryos from heterozygote in-crosses. Injecting embryos with the eGFP-CC mRNA does not result in significant rescue of the mutant phenotype, where an average of 23.2% of the embryos from three independent experiments developed body curvature and cysts, compared to an average of 21.6% of the uninjected embryos. Taken these results together, we conclude that the coiled-coil domain is necessary but not sufficient for Qilin's function.

Next we systematically deleted the rest of the regions in Qilin, and results suggest that both Qilin without the aspartic acid rich region (eGFP-ΔAR) and Qilin without the C-terminal tail (eGFP-ΔCT) can still rescue mutant phenotypes, where an average of 3.2% and 1.0% of the embryos injected with the respective mRNA developed curvature and cysts. These results suggest that the aspartic acid rich domain, as well as the C-terminal tail, is dispensable for Qilin's function.

Lastly we tested the rescuing ability of mRNA encoding both the N-terminal region and the coiled-coil domain (eGFP-NT+CC), and we discovered that an average of 3.6% of the heterozygous in-cross embryos injected with this mRNA developed body curvature and cysts, compared to an average of 30.0% of the embryos developed these phenotypes in uninjected siblings. These results, combined with the observation that neither the coiled-coil domain nor the N-terminal domain alone can significantly rescue mutant phenotypes, lead us to conclude that the N-terminal tail and the coiled-coil domain together are sufficient for the function of Qilin.

## Discussion

### Qilin in vertebrate cilia biogenesis and maintenance

Qilin is a novel protein we isolated in a previous genetic screen for cystic kidney mutants in zebrafish [12]. Consistent with the critical role of cilia in kidney cyst formation, Qilin was subsequently linked to cilia. In the nematode *C. elegans*, mutants of *qilin*'*s* homologue *dyf-3* show truncated cilia projections [21]. In addition, *dyf-3* transcription is regulated by DAF-19, a transcription factor that regulates multiple ciliary genes in *C. elegans* [29], [30]. In the green alga *Chlamydomonas*, Qilin is present in the flagellum proteome and the expression of *qilin* is highly up regulated during flagellum regeneration[23]. In this study, for the first time in an in vivo vertebrate system, we provide clear evidence that Qilin plays an essential role in both cilia biogenesis and maintenance.

We showed that *qilin^3959A^* mutants display cilia biogenesis defects in multiple cell types, including MCCs in the pronephric duct, photoreceptors of the eye, and hair cells in the lateral line organ. Further, the maternal contribution of *qilin* and the differential development timing of cilia biogenesis allowed us to uncover Qilin's role in cilia maintenance. Specifically, we found that single cilia and bundled cilia in the pronephric duct form at different developmental stages: while single cilia can be detected as early as the 24 somite stage, bundled cilia are not visible in a majority of embryos until 30 hpf. Interestingly, in *qilin^3959A^* mutants, single cilia initially assemble normally, but degenerate as embryos develop, and by 5 dpf single cilia are no longer visible in mutant embryos. By contrast, bundled cilia never form. One possible interpretation is that Qilin's function is cell type specific. However, given that *qilin* transcript is maternally supplied, we postulate that maternally expressed Qilin is able to support the biogenesis of single cilia in the pronephric duct and the gradual degradation of the maternal protein reveals Qilin's role in cilia maintenance. The failure of cilia formation in photoreceptors in the eye and hair cells in the lateral line organ, both of which form single cilia later in development, supports our hypothesis.

Interestingly, the phenotype of the *qilin* mutant we observed is different from previously observed in a *qilin* morphant [31]. It is possible that the published oligo was able to block the translation of maternally deposited *qilin* mRNA, thus revealing more severe phenotypes. Alternatively, off-target effect of the morpholino oligo, which is different from the one used in this study, could contribute to the reported morphant phenotypes. The generation and analysis of maternal zygotic *qilin* mutants in the future should be able to provide definitive results to distinguish between these two possibilities.

### Qilin and IFT

In this study, we show that partial reduction of Qilin synergize with partial reduction of IFT B complex components, suggesting that *qilin* genetically interacts with IFT B complex genes. Further, despite the similar morphological phenotypes displayed by Group II mutants isolated from our previous mutagenesis screen for cystic mutants, careful analysis revealed distinct ciliary defects in these mutants. For example, In *scorpion/arl13b* mutants, pronephric cilia are severely shortened and reduced in number at 50 hpf, but a significant number of cilia manage to form by 5 dpf [24]. In *seahorse* mutants, neither cilia density nor length is significantly different from those in wild type embryos [26]. Interestingly, both *ift57* and *ift172* mutants show cilia maintenance defect in SCCs of the pronephric duct and cilia biogenesis defect in MCCs of the pronephric duct, photoreceptors and the lateral line organ [25]. The almost identical cilia biogenesis and maintenance defects in *qilin* and IFT B-complex mutants suggest that Qilin's function is more closely associated with IFT B complex, among other cilia-associated proteins.

This hypothesis is also supported by other studies. Qilin homolog was seen to travel in the cilium in a pattern and velocity precisely as those observed for the anterograde movement of IFT particles and IFT motors in *C. elegans* [19], [23]. Further, in *C. elegans*, loss of BBSome leads to the separation of the IFT-B/OSM-3 complex and the IFT-B/OSM-3 complex. Importantly, under this condition, the Qilin homologue travels specifically with the latter, providing strong support for the association of Qilin with IFT B complex [32]. Finally, Qilin was pulled down together with Ift27, Ift57 and Ift172 from zebrafish embryo lysate [33]. Whether Qilin is a core component of IFT B complex or it functions as a peripheral regulator of this complex remains unclear at this stage. Biochemical purification of IFT complexes, as those done in *Chlamydomonas* [34], in both zebrafish and *C. elegans*, will provide direct evidence for the role of Qilin in IFT complexes.

### Structural features of Qilin protein

Similar to many cilia associated proteins, structure of Qilin reveals little regarding its function. The only recognizable domain in Qilin is a coiled-coil domain followed by an aspartic acid rich region in the middle of the protein. Through serial deletion analysis, we showed that the N-terminal region and the coiled-coil domain are both required for Qilin's function in normal body axis and kidney development, while both the aspartic acid-rich region and the C-terminal tail are dispensable for these functions. This result is consistent with our observation that the N-terminal region and the coiled-coil domain are highly conserved between multiple species, whereas the aspartic acid-rich region and the C-terminal end of the protein are less well conserved. Identification of proteins that directly interact with the N-terminal region and the coiled-coil region of Qilin will provide critical insight to mechanisms underlying Qilin's function.

## Supporting Information

Table S1 **Sequence conservation of Qilin protein. **The names and accession numbers of Qilin's homologue in *C. elegans*, *Drosophila Melanogaster*, *Mus musculus*, and *Homo sapiens* are listed. The percentages of protein sequence identity of the different homologues to zebrafish Qilin are also listed.
(PDF)
