# Supplementary material for: Qilin Is Essential for Cilia Assembly and Normal Kidney Development in Zebrafish
Source: PLoS One. 2011 Nov 15;6(11):e27365. doi: 10.1371/journal.pone.0027365 (PMC3216947; doi:10.1371/journal.pone.0027365)
Supplement: Table S1 — Sequence conservation of Qilin protein. The names and accession numbers of Qilin's homologue in C. elegans, Drosophila Melanogaster, Mus musculus, and Homo sapiens are listed. The percentages of protein sequence identity of the different homologues to zebrafish Qilin are also listed. (PDF) [file pone.0027365.s001.pdf]

Table S1. Sequence conservation of Qilin protein.

Species	Name	Accession Number	sequence identity to zebrafish Qilin
C. elegans	*dyf3*	Q6I6D5.1	33%
Drosophila melanogaster	*CG17599*	Q9VR64	25%
Mus musculus	*cluap1*	Q3TM98	64%
Homo sapiens	*CLUAP1*	Q96AJ1.4	62%
